# Golden hour management in the patient with intraparenchymal cerebral hemorrhage: an Italian intersociety document

**DOI:** 10.1186/s44158-025-00244-z

**Published:** 2025-05-09

**Authors:** Gianluigi Morello, Daniela Alampi, Raffaele Aspide, Alessandra Beretta, Rita Bertuetti, Federico Bilotta, Etrusca Brogi, Giovanni Buscema, Anselmo Caricato, Davide Caruzzo, Carlo Alberto Castioni, Arturo Chieregato, Andrea Cortegiani, Alessandro De Cassai, Andrea Fabbri, Domenico Gelormini, Paolo Gritti, Lucrezia Guadrini, Alberto Librizzi, Nicola Latronico, Nicola Limbucci, Marina Munari, Edoardo Picetti, Giuseppina Pipitone, Gianluca Pucciarelli, Chiara Robba, Danilo Toni, Salvatore Sardo, Simone Maria Zerbi, Nicola Zugni, Frank Rasulo

**Affiliations:** 1 ASP Catania, Anesthesia and Intensive Care, Militello Hospital, Catania, Italy; 2 Unit of Anesthesia, Department of Clinical and Surgical Translational Medicine, Intensive Care and Pain Medicine, Sant’Andrea Hospital, Sapienza University, Rome, Italy; 3 IRCCS Istituto delle Scienze Neurologiche di Bologna, Anesthesia and Intensive Care Unit, Bologna, Italy; 4 Neurocritical and Postoperative Care, Neuroanesthesia, ASST Spedali Civili Di Brescia, Brescia, Italy; 5 Department of Anesthesiology and Intensive Care Medicine, University of Rome “La Sapienza”, Rome, Italy; 6 Neuroscience Intensive Care Unit, ASST Grande Ospedale Metropolitano Niguarda, Milan, Italy; 7 Department of Anesthesia and Intensive Care Unit, Azienda Ospedaliero Universitaria Policlinico“G. Rodolico — San Marco”di, Catania, Italy; 8 Terapia Intensiva Neurochirurgica, Fondazione Policlinico Universitario A.Gemelli IRCCS, Rome, Italy; 9 Università Cattolica del Sacro Cuore, Rome, Italy; 10 Azienda Sanitaria Universitaria Friuli Centrale Di Udine, Udine, Italy; 11 Department of Precision Medicine in Medical, Surgical and Critical Care (Me.Pre.C.C.), University of Palermo, Palermo, Italy; 12 Department of Anesthesia, Intensive Care and Emergency, University Hospital Policlinico Paolo Giaccone, AnalgesiaPalermo, Italy; 13 University of Padua, Padua, Italy; 14 Institute of Anesthesia and Intensive Care, University Hospital of Padua, Padua, Italy; 15 Emergency Department, Local Health Agency of Romagna, Forlì, FC Italy; 16 Neurocritical and Postoperative Care Unit, Department of Emergency, Anesthesia and Intensive Care, Azienda Ospedaliero Universitaria Integrata Di Verona, Verona, Italy; 17 Department of Anesthesia and Intensive Care Unit, Papa Giovanni XXIII Hospital, Bergamo, Italy; 18 Department of Medical and Surgical Specialties, Radiological Sciences and Public Health, University of Brescia, Brescia, Italy; 19 Department of Emergency, Spedali Civili University Hospital, Brescia, Italy; 20 Interventional Neurovascular Unit, Careggi University Hospital, Florence, Italy; 21 Department of Anesthesia, Intensive Care and Neurointensive Care, University Hospital of Padua, Padua, Italy; 22 Department of Anesthesia and Intensive Care, Parma University Hospital, Parma, Italy; 23 Department of Neurosurgery, Parma University Hospital, Parma, Italy; 24 Department of Biomedicine and Prevention, University of Rome Tor Vergata, Rome, Italy; 25 Department of Surgical Science and Integrated Diagnostic, University of Genova, Genoa, Italy; 26 IRCCS Ospedale Policlinico San Martino, Genoa, Italy; 27 Emergency Department Stroke Unit, Department of Human Neurosciences, Sapienza University of Rome, Rome, Italy; 28 Department of Medical Sciences and Public Health, University of Cagliari, Monserrato, Italy; 29 Department of Emergency, Anesthesia and Intensive Care, Neurointensive Care, ASST Lariana Ospedale Sant’Anna, San Fermo Della Battaglia (CO), Como, Italy; 30 Dept. of Neuroanesthesia, Neurocritical and Postoperative Care, ASST Spedali Civili University Affiliated Hospital of Brescia, Brescia, Italy

**Keywords:** Intracerebral hemorrhage, Clinical questions, Computed tomography angiography, Glasgow Coma Scale, Systolic blood pressure, Tranexamic acid, Electroencephalogram

## Abstract

**Background:**

Spontaneous intracerebral hemorrhage (ICH) accounts for 9–27% of all strokes worldwide and is associated with high mortality and disability. The main causes include vascular malformations, small- and large-vessel angiopathies, and coagulation disorders. Mortality rates reach approximately 40% at 1 month and 54% at 1 year, largely influenced by early management decisions. Rapid intervention, particularly within the first hour, is crucial, especially for patients initially treated in peripheral hospitals. This consensus document, developed by SIAARTI with the endorsement of multiple medical societies, aims to standardize ICH management based on hospital capabilities, aligning with the “time is brain” principle and the 2022 AHA guidelines.

**Methods:**

A multidisciplinary panel of experts—including neurointensivists, neuroanesthesiologists, neurologists, neuroradiologists, emergency physicians, and neuroscience nurses—developed this consensus document. The process combined a systematic literature review with a modified Delphi method, prioritizing clinical questions using the UCLA-RAND appropriateness methodology. Literature searches were conducted on PubMed following PRISMA 2020 guidelines. Statements were formulated based on both evidence and expert consensus, and the final document underwent external peer review.

**Results:**

Computer tomography (CT) angiography, with over 90% sensitivity and specificity, is a key tool for identifying macrovascular abnormalities and detecting active bleeding, a critical factor in poor outcomes. Prognostic models, such as the ICH score, assist in clinical decision-making. Strict blood pressure control (target 130–140 mmHg) and early intubation in appropriate cases help mitigate hematoma expansion. Anticonvulsants are recommended only for patients with documented seizures. In cases of anticoagulant-related hemorrhage, prothrombin complex concentrates are effective for rapid reversal, though their long-term impact remains uncertain. Intensive care unit (ICU) admission is determined by ICH severity, with severe cases benefiting from specialized neurocritical care.

**Conclusion:**

A multidisciplinary and inter-societal discussion provided key recommendations for the immediate management of ICH, based on the available literature. While only a few topics are supported by robust evidence, experts strongly recommend early brain angio CT, risk stratification using scoring systems, clear communication of patient data, and intubation for impaired consciousness. Blood pressure should be controlled with alpha- and beta-blockers, avoiding hypotension. Anticoagulant reversal should be appropriately managed, and eligible patients should be centralized in ICU and neurosurgical centers using dedicated scoring systems.

## Background

Spontaneous intracerebral hemorrhages (ICH) are a significant cause of stroke, accounting for 9 to 27% of strokes worldwide. This condition arises from a variety of factors, including micro- and macroangiopathy, vascular malformations, and coagulation disorders.

ICH is associated with a high mortality rate: nearly 40% of patients die within a month, with up to 54% succumbing within a year. The decision to withhold treatment, often due to ethical considerations about the appropriateness and proportionality of care, can further impact these rates. Survivors often experience significant disabilities that negatively affect their quality of life. Thus, optimizing patient care and management strategies is essential to mitigate both mortality and morbidity in ICH cases [1].

Early intervention within the first hour of diagnosis is crucial, particularly since many patients initially receive care in hospitals without neurology or neurosurgery units. Therefore, it is imperative to swiftly diagnose and treat these patients to improve outcomes.

This document, authored by the Italian Society of Anesthesiology, Resuscitation, and Intensive Care (SIAARTI) and co-endorsed by several medical societies, outlines a standardized approach tailored to the specific capabilities of hospitals, whether they function as “hubs” or “spokes” in this context. The protocol underscores the “time is brain” concept, advocating for expedited interventions within the first 24 h following diagnosis and stabilization, in accordance with the 2022 guidelines from the American Heart Association (AHA) [2].

## Methodology

The steering committee, comprising G. M. and F. R., assembled a multifaceted panel of experts renowned for their expertise in ICH and stroke management. The panel consisted of neurointensivists, neuroanesthesiologists, neurologists, neuroradiologists, emergency physicians, and neuroscience nurses. A methodologist (C. R.) was assigned to the project, and two additional experts (A. C., A. Ch.) were included in the advisory committee (A. C.). In addition to members from SIAARTI, representatives from several medical societies were invited, including the Society of Medical and Interventional Radiology (SIRM), National Association of Neuroscience Nurses (ANIN), Italian Society of Neurology (SIN), Italian Society of Emergency Medicine (SIMEU), and Italian Stroke Association-Italian Ictus Association (ISA-AII). These collaborations aimed to ensure a comprehensive and multidisciplinary approach.

The methodology employed a blend of systematic literature review complemented by a modified Delphi process, prioritizing clinical questions using the UCLA-RAND appropriateness methodology [3]. Literature searches were conducted on PubMed following PRISMA 2020 guidelines [4].

### Clinical trial number

This is not applicable.

## Results

The results are described as individual paragraphs related to the nine items. Table 1 summarizes the queries, with the related subcategories and the related expert panelists’ answers.

**Table 1:** This table contains the nine main statements and their sub-statements and related expert panel indications. The last column shows the percentages of agreement resulting from the votes

	Statement		Percentage of agreement
1. Indications and/or criteria for performing CT angiography	1.a	The expert panel considers CT angiography appropriate in patients younger than 70 years old and spontaneous lobar intracerebral hemorrhage	100% (*IQR* 7–9)
	1.b	The expert panel considers CT angiography appropriate in patients younger than 45 years old and spontaneous deep intracerebral hemorrhage or posterior fossa hematoma	100% (*IQR* 7–9)
	1.c	The expert panel considers CT angiography appropriate in patients aged between 45 and 70 years old with no clinical history of systemic arterial hypertension, in cases of spontaneous deep intracerebral hemorrhage or posterior fossa hematoma	100% (*IQR* 7–9)
	1.2	The expert panel considers CT angiography appropriate to exclude cerebral venous thrombosis	96.15% (*IQR* 7–9)
	1.3	The expert panel considers early CT angiography appropriate to identify patients at risk of hematoma expansion	96.15% (*IQR* 7–9)
2. What is the rationale for using scores for prognostic stratification (e.g., ICH score)	2.1	Stratification of patients at admission using a severity score is indicated	100% (*IQR* 7–9)
	2.2	The use of prognostic scoring model that can predict outcome is useful; however, they cannot be used as the sole criterion for deciding the intensity of treatment and/or its withdrawn	100% (*IQR* 7–9)
3. What are the criteria for indicating orotracheal intubation?	3.1	Orotracheal intubation is indicated in the following: •In patients with a severe impairment of the level of consciousness (a score of less than 8 on the *GCS* ≤ 8) •In case of dysfunctional airway-protective mechanisms and/or inadequate gas exchange	100% (*IQR* 7–9)
4. What are the systolic BP targets to maintain?	4.1	In hemorrhagic stroke with BP 150–220 mmHg, start prompt treatment targeting 130–140 mmHg, avoiding rapid shifts, hypotension, and hypoperfusion	100% (*IQR* 7–9)
	4.2	In patients with hemorrhagic stroke and systolic *BP* > 220 mmHg, a BP reduction of approximately 90 mmHg is advisable	88.5% (*IQR* 7–9)
	4.3	In severe hemorrhagic stroke (GCS alteration or hematoma > 30 ml), BP targets should be adjusted based on *CPP* ≥ 60–70 mmHg when available	88.5% (*IQR* 7–9)
	4.4	For the hypertension, drugs with rapid onset and short half-life are preferable	100% (*IQR* 7–9)
5. The rationale for utilizing invasive and continuous blood pressure monitoring in patients with intraparenchymal cerebral hemorrhage	5.1	Invasive blood pressure monitoring is recommended as early as possible in patients with impaired consciousness	92.3% (*IQR* 7–9)
	5.2	In patients with a large intracerebral hematoma for whom continued treatment is considered beneficial, invasive blood pressure monitoring is advisable	96.15% (*IQR* 7–9)
	5.3	Invasive blood pressure monitoring is recommended in patients with atrial fibrillation and hemodynamic instability	88.5% (*IQR* 7–9)
6. What is the rationale for the use of tranexamic acid within the first hour of diagnosis?	6.1	There is no clear evidence to support the use of tranexamic acid in patients with intraparenchymal cerebral hemorrhage	96.15% (*IQR* 7–9)
7. What is the role of seizure prophylaxis in the first hour after diagnosis?	7.1	Expert panelists recommend against seizure prophylaxis in patients with ICH in the absence of epileptic seizures (observed clinically and/or with EEG)	100% (*IQR* 7–9)
8. What is the role of early use of antagonists in patients on anticoagulant or antiplatelet?	8.1.a	Expert panelists recommend early reversal of anticoagulant drugs in patients with ICH on anticoagulant therapy	100% (*IQR* 7–9)
	8.1.b	Expert panelists recommend reversal of anticoagulant drugs with specific antagonists whenever possible, or with 3–4 factors prothrombin complex concentrates, instead of fresh-frozen plasma	100% (*IQR* 7–9)
	8.2.a	Expert panelists recommend reversal of antiplatelet therapy, particularly aspirin, with platelet administration, only in ICH patients eligible for urgent neurosurgical intervention	100% (*IQR* 7–9)
	8.2.b	Expert panelists recommend against the administration of platelets or other platelet-active drugs (e.g., desmopressin) as early reversal in patients on antiplatelet therapy not undergoing urgent neurosurgical intervention	96.15% (*IQR* 7–9)
9. Admission criteria for intensive care (general or neuro, with or without neurosurgery)	9.1	As the need for intensive care relies upon the degree of vital functions’ impairment, expert panelists recommend that patients with an initial GCS score < 12–13, systolic blood pressure > 160 mmHg, volume of bleeding > 15–20 ml, intraventricular hemorrhage, and subtentorial location of the hemorrhage should be admitted in an intensive care unit, making use of multidisciplinary expertise	96.15% (*IQR* 7–9)
	9.2	Expert panelists recommend that patients with intraparenchymal cerebral hemorrhage and neurological impairment, intraventricular bleeding, hydrocephalus, or subtentorial location of the hematoma should be better referred to a specialized neurointensive care unit	100% (*IQR* 7–9)
	9.3	Expert panelists recommend that in case of devastating brain injury, with poor neurological prognosis, ICU admission for organ donation purposes (DBD or DCD) is appropriate and should be pursued especially in the presence of a willingness to donate organs (expressed by the patient in life or reported by family members)	100% (*IQR* 7–9)

*CT* Computer tomography, *GCS* Glasgow Coma Scale, *BP* Blood pressure, *CPP* Cerebral perfusion pressure, *EEG* Electroencephalogram, *DBD* Donation after brain stem death, *DCD* Donation after circulatory death

### Statement 1: Indications of CT angiography

Computed tomography (CT) angiography has demonstrated excellent accuracy in the detection of macrovascular anomalies, with sensitivity and specificity levels exceeding 90% [5–9]. The “DIagnostic AngioGRAphy to find vascular Malformations” (DIAGRAM) score highlighted that factors predicting intraparenchymal cerebral hemorrhage due to macrovascular disease include young age, lobar or posterior fossa hemorrhages, and the absence of small vessel disease [10]. The predictive probability of macrovascular disease was approximately 1% of patients aged 50 to 70 years with deep cerebral hemorrhage and small vessel disease. The probability increased to over 50% in patients aged between 18 and 50 with lobar or posterior fossa ICH without small vessel disease. Furthermore, Wilson et al. found that negative CT angiography (CTA) associated with small vessel disease and a positive history of arterial hypertension has a low possibility of an underlying macrovascular etiology (1.8%) [11].

Only 30% of cerebral venous thrombosis can be recognized with a non-contrast CT scan, with the visualization of a hyperdensity sign in correspondence to the venous sinuses [12]. Contrast-enhanced computed tomography can improve diagnostic accuracy. This technique has shown a sensitivity of 95% and a specificity of 91% with an overall precision between 90 and 100%, depending on the vein or venous sinus studied.

CTA allows the identification of active bleeding (“spot sign”) through the visualization of the arterial extravasation of the contrast agent predicting further growth of the hematoma. The presence of spot signs is associated with a worse ICH score, lower Glasgow Coma Scale (GCS) at emergency room arrival, larger hematoma size, higher rate of expansion, and increased 90-day mortality. Furthermore, the presence of spot signs is correlated with a longer intensive care unit (ICU) stay and overall hospitalization [13–15].

### Statement 2: Rationale for using prognostic scoring model

Prognostic scores such as the ‘ICH score” ([16–18]) aid in assessing both the risk of death and functional outcomes for patients with ICH. Indeed, some authors believe that the predictive capacity of treating physicians is superior to the use of scores [19]. This phenomenon can be probably due to the fact that the scores are necessarily based on few covariates [20, 21] and affected by the self-fulfilling prophecy bias due to withdrawal of care, except for the maximally treated (MAX)-ICH score [22, 23].

### Statement 3: Indications for orotracheal intubation

While there is a lack of trial evidence supporting superior breathing management with tracheal intubation in ICH patients [24, 25], consideration should be given to neurological impairments such as altered cranial nerve, breathing disorders, and vomiting, all of which can lead to airway collapse and hypoxemic events impacting intensive care outcomes. Evaluations of neurological status, airway protection mechanisms, and gas exchange episodes guide the decision toward orotracheal intubation, accounting for proportionality related to patient age, preexisting conditions, and residual lifetime.

### Statement 4: What are the systolic blood pressure targets to maintain?

In hemorrhagic stroke, early blood pressure (BP) management is recommended to the target value of 130–140 mmHg for ≥ 7 days to limit hematoma expansion and neurological decline, extending over 7 days if rebleeding risk persists [26, 27].

A meta-analysis published in 2021, including 16 RCTs with a total number of 5895 patients, found that the treatment of systolic blood pressure (SBP) values, in the first 7 days after the acute event, does not significantly modify the functional outcome at 3–6 months but can influence the volumetric increase of the intracerebral hematoma in patients with hemorrhagic stroke [28]. Conflicting results came from the Intensive Blood Pressure Reduction in Acute Cerebral Hemorrhage Trial (INTERACT)−2 and the Antihypertensive Treatment of Acute Cerebral Hemorrhage (ATACH)-II randomized trials [29].

It is advisable to start pressure treatment within 2 h of the onset of neurological symptoms, with the aim of reaching the BP target within 1 h of starting therapy [2]. The aim of “INTERACT-4” trial was to evaluate the efficacy of urapidil in the prehospital environment for the hyperacute treatment of hypertensive crisis [30].

Patients with hemorrhagic stroke have a higher acute kidney injury (AKI) risk [31], especially in large ICH, due to multiple factors (i.e., chronic hypertensive nephropathy, hypovolemia). In those without prior nephropathy, AKI is linked to increased mortality [32]. However, it is not yet clear whether AKI is directly responsible for this increased mortality or whether it is more a marker of the gravity of the disease from the onset [33].

In patients with moderate-severe intracerebral hemorrhage (*GCS* < 13, National Institutes of Health Score (NIHSS) ≥ 10, ICH volume ≥ 30 ml, or intraventricular hemorrhage (IVH)) and with consequent risk of intracranial hypertension, intensive BP treatment (*SBP* 110–140 mmHg) is associated with a lower frequency of hematoma expansion without reducing mortality or disabilities [34]. Indeed, a reduction in BP can lead to an increase in the cerebral blood volume and therefore according to the compliance of intracranial pressure.

Although there is no specific recommendation regarding the choice of the antihypertensive drug, rapid-onset and short-lived drugs with any possibility of continuous infusion administration allow to reduce excessive SBP fluctuations. At the same time, it is better to avoid drugs with a venodilator effect due to their unfavorable effect on the hemostasis mechanism and on the cerebral perfusion pressure (CPP). A better functional outcome has been highlighted for patients treated with alpha- and beta-blockers (i.e., urapidil and labetalol): this effect can be partially explained by the ability of these drugs to act particularly on the arteriolar compartment of peripheral resistance and mitigate the sympathetic response [35].

### Statement 5: The rationale for utilizing invasive and continuous blood pressure monitoring in patients with intraparenchymal cerebral hemorrhage

BP control is crucial to limit hematoma growth. Noninvasive monitoring underestimates the SBP and overestimates diastolic blood pressure; the discrepancy between the two methods tends to increase in patients with high values of SBP [36]. In all these cases, invasive BP monitoring can be indicated. In large ICH (> 30 ml) or IVH requiring an external ventricular drain (EVD), invasive BP monitoring is essential to optimize CPP [37].

In patients with atrial fibrillation (AF), there is a high variability of pressure measurement in different points of the arterial tree [38]. In the patient with AF, the noninvasive measurement of the BP can be used for cardiac frequency values < 90 bpm or reduced variability of the heart rate (< 10 bpm difference in three subsequent beats) [39]. In the case of high values or marked variations in the time of heart rate, especially if associated with changes in BP, invasive monitoring can be recommended [40].

### Statement 6: What is the rationale for the use of tranexamic acid within the first hour of diagnosis?

There are no specific trials available that clarified the effect of the use of tranexamic acid (TXA) in patients with ICH. Even if the use of TXA seems to reduce the risk of hematoma expansion, there is no evidence available showing a neurological outcome improvement and/or a reduction in mortality.

Overall, the studies examined report often contrasting results. The Tranexamic acid for hyperacute primary IntraCerebral Haemorrhage (TICH-2) trial was a controlled randomized study that included 2345 patients aimed to compare placebo versus TXA. The authors found that the reduction of hematoma expansion in patients treated with TXA was not associated with a reduction in mortality or at modified Rankin score (mRS) disability [41]. A sub-analysis of the TICH-2 trial confirmed that TXA is able to reduce the expansion of the hematoma in the first 24 h, although this effect is only partially associated with neurological outcome [42].

A post hoc study of the “STOP-AUST” trial failed to find any significant reduction in hematoma growth after the administration of TXA in patient with IVH [43].

### Statement 7: What is the role of seizure prophylaxis in the first hour after diagnosis?

In patients with ICH without evidence of detectable seizures, both clinically or instrumentally, the anticonvulsant prophylaxis has not shown any improvement in outcome, reducing the risk of early and late seizures or decreasing mortality [44–49]. Despite most published studies are predominantly uncontrolled and there remains some uncertainty regarding the definition and terminology of some electroencephalogram (EEG)-detected alterations and the choice of anti-epileptic drugs, the panel does not suggest any prophylaxis if there are no detectable seizures, clinically or instrumentally (preferably monitored with 24-h EEG continuous monitoring). Conversely, in patients with evidence of clinical or subclinical epileptic seizures, and/or detected by EEG, authors promote starting a dedicated anti-epileptic therapy.

In uncertain cases, such as an unexpected altered state of consciousness, not otherwise justifiable, in comatose patients and not clinically explorable, or in patients with lobar hemorrhagic lesions and/or near the cortex (1 cm) or in epileptogenic areas [50], a prolonged EEG (> 24 h) should be performed to check for possible epileptogenic foci or nonconvulsive disease state (NCSE).

Authors found the cortical involvement, age, hemorrhage volume, and acute symptomatic seizure (CAVE score) to be useful in identifying those patients at increased risk for late seizures [51]. Should the EEG show suggestive signs, an appropriate prophylaxis or anti-epileptic therapy can be started; any change of therapy should be based on neurophysiological control [52]. A recent trial has shown that the use of levetiracetam could be effective in reducing the risk of seizures during the early phases in patients with ICH [53].

### Statement 8: What is the role of early use of antagonists in patients on anticoagulant or antiplatelet?

The administration of three- or four-factor prothrombin complex concentrates (PCC) ensures the rapid international normalized ratio (INR) reversal in a high percentage of patients on dicumarol therapy (62.9 to 80%, > 96 h) [54–58]. Nonetheless, INR reversal alone might not be enough to influence functional outcome or mortality [59].

A randomized study comparing fresh-frozen plasma, vitamin K, and PCC showed that PCC was able to improve overall survival compared to other therapies, although with similar disability scores [60]. In patients on oral anticoagulant therapy, early reduction of *INR* < 1.3 was associated with a lower expansion of the hematoma [61]. Moreover, the use of PCC 3 or 4 factors is associated with increased stability in hematoma volume stability in both patients on rivaroxaban and apixaban [62–65].

Andexanet alfa, a specific antidote, has demonstrated an excellent hemostatic response (82–90.9%) with a low incidence of thrombotic events (10%) in several studies [66–68], also confirmed by a post hoc analysis of the andexanet alfa, a novel antidote to the anticoagulation effects of factor Xa inhibitors (ANNEXA)−4 study, which showed that the hemostatic efficacy was 93.8% for apixaban and 92.6% for rivaroxaban, with 9.3% of thrombotic events in the cohort [69]. Direct comparison of andexanet alfa with four-factor PCC in non-randomized retrospective studies revealed superior [70] or at least comparable [71] results in terms of hematoma stability, functional outcome, and risk of thrombotic events.

Idarucizumab showed to be effective in preventing hematoma expansion and in improving the outcome [72] in patients who were treated with dabigatran; however, a registry study conducted in the USA did not confirm this result [73]. Other studies suggested a good hemostatic effect (79–89%) after administration of factor eight inhibitor bypassing activity (FEIBA), as highlighted by the stability of the hemorrhage volume in patients with cerebral hemorrhage treated with apixaban or rivaroxaban, whether they have undergone surgery or not [62, 74, 75].

The usefulness of platelet administration, in patients on antiplatelet therapy, has been investigated by several studies. However, only a few of them have shown efficacy in terms of reduced hematoma expansion, better outcomes, and mortality reduction [76–78]. Most of the literature available to the panel demonstrates a lack of efficacy in those outcomes [79–81]. Some studies showed a higher risk of either death or an unfavorable neurological outcome related to platelet administration [82]. The use of desmopressin alone [83] or in combination with platelet administration [80] has also not been found effective in improving the outcome of patients with intracranial hemorrhage.

### Statement 9: Admission criteria for intensive care (general or neuro, with or without neurosurgery)

Independent predictive factors for intensive care admission after ICH have been identified, while predictive scores have been developed and validated. In supratentorial hemorrhage, a retrospective analysis in the Klaas study identified the variables related to the indications of intensive care admission and developed a score to facilitate triage, the ICH triage score, which considers three variables: volume of bleeding > 30 ml, GCS score < 13, and intraventricular bleeding. The presence of one or more of these variables supports the indication for intensive care admission (sensitivity of 94.3%, *AUC* = 0.88; *p* < 0.001) [84].

The Intensive Care Triaging in Spontaneous Intracerebral Hemorrhage (INTRINSIC) score is another system of assessment with a total score of 0–9 based on four factors, such as SBP, GCS score, the volume of bleeding, and the presence of intraventricular blood at the entrance [85]. A score > / = 2 in the emergency room (ER) is predictive for ICU admission, while patients with a score of 0 or at least < 2 in the ER are more likely treated, even for the period thereafter, outside of the ICU.

Patel’s work provides an easily calculable score, useful for efficient patient stratification but even to address centralization in those hospitals with the neurosurgery and for the admission to intensive care. The score includes variables such as the GCS score lower than 13 at admission, IVH, subtentorial bleeding, antiaggregant therapy, and the presence of arteriovenous malformation at CTA. The score pragmatically excludes the volume of bleeding as a predictor because its evaluation can be time-consuming in emergency conditions [86].

Patients with intraparenchymal cerebral hemorrhage presenting hydrocephalus, intraventricular bleeding, or infratentorial hemorrhage should be managed in facilities where a neurosurgical ICU, neuro specialist ICU, or a general ICU with neurosurgical support is available. Those conditions relate to better outcomes and reduced mortality [87, 88].

The risk of death of patients with higher GCS score, younger age, larger volume hematomas and female sex who are transferred to hub hospital, adjusted for established prognostic factors for cerebral hemorrhage, is less than half compared to those who remain in the first reference hospital [89].

Literature data [90] supports the recommendations of the European Stroke Organisation to treat patients with cerebral hemorrhage in a stroke unit or neurocritical care unit (NICU) and suggests that more critical patients may benefit further from treatment in the NICU. However, these recommendations are based on experts’ opinions [1].

## Discussion and up-to-date insights

ICH, a significant neurological emergency, remains associated with substantial mortality and disability rates. Effective management demands a multifaceted strategy, combining prompt diagnosis, prognostic evaluation, and targeted therapies. This report by the expert panel synthesizes consensus-based, widely accepted principles from the literature to guide clinicians in the initial treatment of patients with intraparenchymal cerebral hemorrhage where possible. It offers practical recommendations on CTA utilization, BP control, endotracheal intubation criteria, hemostatic therapy use, and intensive care monitoring practices.

### Contrast neuro-imaging scoring

Rapid evaluation is critical for acute management and prognosis in patients with ICH. CTA is pivotal in the early detection of vascular anomalies and assessment of hematoma growth risk, aiding in differential diagnosis. Several research groups contribute to developing scoring systems, notably the DIAGRAM score for macrovascular cause probability prediction and the Causal CLASsification system for ICH Subtypes (CLAS-ICH) system for nuanced subtype classification [91].

### Predictor score

Crucially, scoring systems like the ICH score [16] and MAX-ICH [22] enhance stratification, aiding treatment optimization. The FUNC score was also proposed with the aim of mainly evaluating the functional outcome [92]. Cai et al.’s primary brainstem haemorrhage (PBSH) score provides valuable predictive insights for brainstem hemorrhages [93]. However, these tools should not dictate sole criteria for care intensity or treatment withdrawal decisions; instead, they optimize resource allocation and personalize patient management.

### Airway management and ventilation

Endotracheal intubation is warranted for patients with significantly impaired consciousness (*GCS* ≤ 8) or absent airway reflexes, aiming to prevent aspiration and stabilize oxygenation and clinical stability. Nonetheless, ICH diagnosis independently correlates with reduced 1-year survival in those intubated acutely (statement 3) [25]. Beyond mortality, poor functional outcomes (measured as a mRS of 4–6) have a very high incidence (66.5%) at 1 year. This raises an important issue of proportionality in care: in patients with severe stroke requiring mechanical ventilation, discussions with caregivers, patients, and their families about post-ICU trajectories and functional outcomes are essential [94].

### Blood pressure management

BP control is vital to mitigate hematoma enlargement (statement 4). Invasive monitoring benefits patients with impaired consciousness, large hematomas, at high risk of AF, or hemodynamic instability, facilitating precise pressure manipulation. The recent INTERACT-4 study from May 2024 published in the *New England Journal of Medicine* underscores prehospital SBP reduction (to 159 mmHg) efficacy in improving neurological outcomes specifically for ICH patients, even though full target BP reduction was not met (140 mmHg) [95].

### Antifibrinolytic therapy

While tranexamic acid has theoretical antifibrinolytic potential, current evidence does not robustly support its routine use in ICH (“Statement 6: What is the rationale for the use of tranexamic acid within the first hour of diagnosis?”). The tranexamic acid for hyperacute onset presentation including mobile stroke units (STOP-MSU) trial, published in the *Lancet Neurology* in June 2024, revealed no beneficial effects on outcomes when administered within 2-h poststroke onset [96].

### Seizure’s prophylaxis

Anticonvulsant prophylaxis is not advised for ICH patients without manifest seizures due to potential side effects and drug interactions [97]. Lekoubou’s August 2024 study leveraging machine learning in predicting late seizures post-ICH highlights the potential of employing advanced analytics for enhanced risk prediction and resource allocation. Their models, applying logistic regression and Light Gradient Boosted Machine algorithms, achieved promising accuracy, with area under the receiver operating characteristic curve values of 0.7051 at 1 year and 0.694 at 5 years [98].

### Patients on antiplatelet drugs

In this cohort, reversing anticoagulation early is paramount to prevent hematoma expansion, often favoring specific antagonists or concentrates over plasma (“Statement 8: What is the role of early use of antagonists in patients on anticoagulant or antiplatelet”). Platelet administration for antiplatelet-treated patients is indicated only in emergent surgical scenarios and generally not elsewhere, also based on recent systematic reviews observing worse outcomes in treated patients [99].

### ICU admission

Admission to the ICU is advisable for unstable ICH patients, considering GCS scores, elevated SBP, intraventricular hemorrhage, or deep brain locations (statement 9). Loggini’s systematic review suggests downgrading low-risk patients from the ICU without worsening outcomes, limiting hospital stays [100]. Moreover, the ICU serves as a resource for organ donation with patient/family consent.

### Limitations

Among the main limitations of this document, it should be noted that it does not constitute formal guidelines, as no grading systems were used for literature analysis, nor was the literature extracted through a systematic review of major research databases. Moreover, it does not include specific or highly specialized elements, as this was not among the objectives of this document; for these aspects, reference should be made to publications focusing on individual treatment components. However, some new insights emerge from the literature published after the drafting of the aforementioned statements.

## Conclusions

This document’s expert panel synthesis offers practical guidance on managing ICH. Only a few recommendations boast strong scientific backing. Panel recommendations encompass early CTA for most ICH cases, strategic use of prognostic scores, selective intubation for consciousness impairment, judicious blood pressure regulation, appropriate anticoagulant reversal, and access to ICU for patients with severe ICH or planning a donation pathway. Expert panelists speak out against the use of tranexamic acid and anti-epileptic prophylaxis without diagnostic evidence of seizures.

## Data Availability

No datasets were generated or analysed during the current study.

## Abbreviations

ICH: Intracerebral hemorrhage
SIAARTI: Society of Anesthesiology, Resuscitation and Intensive Care
AHA: American Heart Association
SIRM: Italian Society of Medical and Interventional Radiology
ANIN: National Association of Neuroscience Nurses
SIMEU: Italian Society of Emergency Medicine
SIN: Italian Society of Neurology
ISA-AII: Italian Stroke Association-Italian Ictus Association
CQs: Clinical questions
PRISMA: Preferred Reporting Items for Systematic reviews and Meta-Analyses
CT: Computer tomography
DIAGRAM: DIagnostic AngioGRAphy to find vascular Malformations
CTA: Computed tomography angiography
GCS: Glasgow Coma Scale
ICU: Intensive care unit
MAX: Maximally treated
BP: Blood pressure
SBP: Systolic blood pressure
INTERACT: Intensive Blood Pressure Reduction in Acute Cerebral Hemorrhage Trial
ATACH: Antihypertensive Treatment of Acute Cerebral Hemorrhage
AKI: Kidney injury
NIHSS: National Institutes of Health Score
IVH: Intraventricular hemorrhage
CPP: Cerebral perfusion pressure
EVD: External ventricular drain
TXA: Tranexamic acid
TICH- 2: Tranexamic acid for hyperacute primary IntraCerebral Haemorrhage
IVH: Intraventricular hemorrhage
EVD: External ventricular drain
AF: Atrial fibrillation
TXA: Tranexamic acid
mRS: Modified Rankin score
TICH: Tranexamic acid for hyperacute primary IntraCerebral Haemorrhage
EEG: Electroencephalogram
NCSE: Nonconvulsive disease state
CAVE score: Cortical involvement, age, hemorrhage volume, and acute symptomatic seizure
PCC: Prothrombin complex concentrates
INR: International normalized ratio
ANNEXA: Andexanet alfa, a novel antidote to the anticoagulation effects of factor Xa inhibitors
FEIBA: Factor eight inhibitor bypassing activity
INTRINSIC: Intensive Care Triaging in Spontaneous Intracerebral Hemorrhage
ER: Emergency room
NICU: Neurointensive care unit
CLAS-ICH: Causal CLASsification system for ICH Subtypes
PBSH: Primary brainstem haemorrhage (PBSH)
STOP-MSU: Tranexamic acid for hyperacute onset presentation including mobile stroke units
